# Supplementary material for: ‘Accumulating harm and waiting for crisis’: Parents’ perspectives of accessing Child and Adolescent Mental Health Services for their autistic child experiencing mental health difficulties
Source: Autism. 2025 Apr 30;29(8):2111–22. doi: 10.1177/13623613251335715 (PMC12255833; doi:10.1177/13623613251335715)
Supplement: sj-pdf-1-aut-10.1177_13623613251335715 – Supplemental material for ‘Accumulating harm and waiting for crisis’: Parents’ perspectives of accessing Child and Adolescent Mental Health Services for their autistic child experiencing mental health difficulties [file sj-pdf-1-aut-10.1177_13623613251335715.pdf]

Access to mental health services for autistic children and young people

Participant Information Sheet
Research Ethics Committee Reference Number: ***23/PSY/046***

You are being invited to take part in a research project. Before you decide if you are happy to take part, it is important for you to understand why the study is being done and what taking part will involve. Please take time to read this information sheet and decide whether or not you would like to take part.

Thank you very much for your time and support. When you are ready, you can begin the survey by clicking on the Start button at the bottom of the page.

1. What is the purpose of the study?

We are interested in finding out more about **your experiences of trying to access NHS Child and Adolescent Mental Health Services (CAMHS) for your autistic child when experiencing mental health difficulties**. We would also like to learn about the biggest challenges in current mental health services for autistic children, and the ways in which you think services can be improved moving forwards.

The study is being conducted by a team of autistic and non-autistic researchers and parents of autistic young people at Liverpool John Moores University and Edge Hill University.

2. Why have I been invited to participate?

You have been invited to take part in this study as you have identified yourself as a **parent/carer of an autistic child of any age** (up to age 18) who has **sought help for mental health difficulties via CAMHS in the UK, within the last 5 years**.

3. Do I have to take part?

No. It is up to you to decide whether or not to take part. If you do not wish to take part, please close the webpage. You can also stop taking part at any time by closing the webpage.

4. What will happen to me if I take part?

You will be asked to complete a short online survey. The survey will take about 15 minutes or longer to fill out, depending on how much you want to tell us. The survey asks about your experiences of seeking and receiving support for your autistic child's mental health difficulties from CAMHS. You will not have to answer any questions you do not want to, and you can end the survey at any time. We will not be asking for any information which will identify who you are.

5. Are there any potential risks in taking part?

We hope that taking part will not involve any risks for you, but some of the questions may be upsetting as they ask about what things have been like for you and your child when they have been experiencing mental health difficulties. You do not have to answer any questions that you do not want to and at the end of the survey there are some links and numbers to support services. As we cannot identify who has filled out a survey, we will not be able to offer individual help or support.

6. Are there any benefits in taking part?

There will be no personal benefit from taking part in this study. However, the information that you share with us could help us to work out how to better support autistic children and young people experiencing mental health difficulties in the future.

7. Payments, reimbursements of expenses or any other benefit or incentive for taking part

There will be no payment or any benefit or incentive for taking part in this study. Unfortunately, we cannot reimburse any expenses you may incur.

8. What will happen to information/data provided?

The information you provide as part of the study is the study data. Any study data from which you can be identified (e.g. from identifiers such as your name, date of birth, audio recording etc.), is known as personal data. Your participation in this study will not involve the collection/use of personal data. Data we collect will be stored safely and securely. Once we have finished the study, we will keep some of the data so we can check the results. You will not be identifiable in any data we collect, and we will write our reports in a way that no-one can work out that you took part in the study.

9. Who is organising the study?

This study is organised by Liverpool John Moores University.

10. Whom do I contact if I have a concern about the study or I wish to complain?

If you have a concern about any aspect of this study, please contact Dr Emma Ashworth, and we will do our best to answer your query. You should expect a reply within 10 working days. If you remain unhappy or wish to make a formal complaint, please contact the Chair of the Research Ethics Committee at Liverpool John Moores University who will seek to resolve the matter as soon as possible:

Chair, Liverpool John Moores University Research Ethics Committee; Email: FullReviewUREC@ljmu.ac.uk; Tel: 0151 231 2121; Research Innovation Services, Liverpool John Moores University, Exchange Station, Liverpool L2 2QP.

11. Data Protection

Liverpool John Moores University is the data controller with respect to your personal data. Information about your rights with respect to your personal data is available from:

https://www.ljmu.ac.uk/legal/privacy-and-cookies/external-stakeholders-privacy-policy/research-participants-privacy-noticeBy asking one of the study team or contacting us using the information below.

12. Contact details

Principal Investigator: *Dr Emma Ashworth*
Member of LJMU staff
LJMU Email address: *E.L.Ashworth@ljmu.ac.uk*
LJMU School/faculty: *School of Psychology, Faculty of Health*

Thank you very much for your time and support. Please start with the survey now by clicking on the Start button below.

---

* *If you have read the information above and agree to take part with the understanding that the data you submit will be processed accordingly, please tick the box below to start.*
If you no longer wish to take part, please close the survey window.

◯ I consent

We are a team of autistic and non-autistic researchers, and parents of autistic young people. We are interested in finding out more about your experiences of trying to access NHS Child and Adolescent Mental Health Services (CAMHS) for your autistic child (of any age) when experiencing mental health difficulties in the UK. We would also like to learn about the biggest challenges in current mental health services for autistic children and young people, and the ways in which you think services can be improved moving forwards.

Please remember that your answers will be anonymous, you can skip any questions that you do not want to answer, and you can end the survey at any time by closing the web browser. You are welcome to complete this survey more than once if you have more than one child.

Part 1

The first few questions ask about you and your child, and your child's mental health.

You can click on the '?' buttons alongside some questions to get more guidance or examples.

How old is your child?

What is your child's gender identity?

- Girl
- Boy
- Transgender girl
- Transgender boy
- Non-binary
- Other
- Prefer not to say

Whereabouts in the UK do you live?

- England
- Northern Ireland
- Scotland
- Wales

Does your child have an autism diagnosis?

QuestionPro

- On a waiting list
- Diagnosed - NHS
- Diagnosed - privately (via NHS funding)
- Diagnosed - privately (self-funded)
- Child self-diagnosed
- Parental suspected diagnosis
- Other

Does your child have any other long-term physical or mental health conditions or other neurodivergence (e.g., ADHD, dyspraxia, OCD)?

What mental health difficulties first led to you/your autistic child seeking help from mental health services?

How do you feel these mental health difficulties impact on your child's daily life?

We realise that sometimes it can be hard to separate a factor linked to your child's autism and a factor linked to their mental health, so we understand that there may be overlap.

QuestionPro

How do you feel these difficulties impact on **your daily life** and the **life of your family?** ?

What kind of school does your child attend?

- ◯ Mainstream
- ◯ Special school
- ◯ Home school
- ◯ flexi-schooling
- ◯ Alternative provision (e.g., PRU)
- ◯ Online schooling
- ◯ Other

Do you think your child's ability to attend or engage with education/school has been impacted by their mental health difficulties? ?

☐ No ☐ Yes (please explain how)

On average, how many days a week can your child attend school?

Number of days

Part 2

The following questions ask about your and your child's experiences of seeking and receiving support for your child’s mental health difficulties from CAMHS.

CAMHS is the name for the NHS services that assess and support young people with emotional or mental health difficulties. The first step to getting help from CAMHS is usually that you will be referred for a CAMHS assessment, often known as a ‘first appointment’.

You can be referred to CAMHS from various sources including your GP, child’s school, or social care services.

Who did you first speak to about your child being referred to CAMHS for their mental health difficulties?

- ○ GP
- ○ School/nursery
- ○ Paediatrician
- ○ Charity/support organisation
- ○ Social care services
- ○ Youth offending team
- ○ Self-referral
- ○ Friend working in the field
- ○ Other

Did you get a referral to CAMHS when you felt your child needed it?

QuestionPro

- Yes
- Not sure
- No (please explain why not)

Did CAMHS agree to assess your child following their referral?

- Yes
- Not sure
- No (please explain why not and what happened next)

How long did it take for you to get a first CAMHS appointment/assessment, following your referral?

- 0-6 months
- 7-12 months
- 13-18 months
- 19-24 months
- More than 2 years
- Not sure

If any, what other support was offered from the NHS while your child was waiting for their first appointment?

Did your child access any non-NHS support (e.g., private healthcare or charities) while waiting for their first appointment?

- Don't know
- No
- Yes (please provide examples)

Part 3

The following questions ask about your thoughts on your child's first appointment with CAMHS.

The 'first appointment' is when your child is first assessed by CAMHS following a referral, rather than the first therapeutic session.

How helpful do you think your child found their first appointment with CAMHS?

First appointment

N/A unable to engage

Please explain your answer, if you would like to.

How involved were you in your child's first appointment?

- Attended with them
- Waited outside
- Did not attend
- Attended instead of them

- [ ] I spoke to CAMHS on the phone
- [ ] Other

Do you think your child felt comfortable/able to explain their mental health difficulties to the CAMHS professionals?

Rating

Don't know

Please explain your answer, if you would like to.

Was your child's autism diagnosis/pending diagnosis disclosed to the CAMHS professional?

- [ ] Yes
- [ ] No
- [ ] Don't know

When was this diagnosis disclosed?

- [ ] At the point of referral
- [ ] Between the referral and the first appointment
- [ ] At the first appointment
- [ ] Not sure
- [ ] Other

Who disclosed this information?

- ◯ The person making the referral
- ◯ A parent
- ◯ My child
- ◯ Not sure
- ◯ Other

Were there changes in how your child was supported following this disclosure?

How were your autistic child's needs (e.g., communication differences, sensory requirements) accounted for during the first appointment? (e.g., sensory equipment, advanced preparation materials/information, separate waiting room, room adjustments)

Did CAMHS agree to provide support for your child after the first appointment? ?

- ◯ Yes
- ◯ Don't know
- ◯ No (please explain why not and where they signposted you to instead)

If yes, what support did they offer?

- ☐ Cognitive Behavioural Therapy (CBT) - one-to-one

- [ ] Cognitive Behavioural Therapy (CBT) - group
- [ ] Dialectical Behaviour Therapy (DBT) - one-to-one
- [ ] Dialectical Behaviour Therapy (DBT) - group
- [ ] Family therapy
- [ ] Drama therapy
- [ ] Art therapy
- [ ] Outdoor therapy
- [ ] Other
- [ ] I wasn't told
- [ ] Don't know

Do you think your child felt comfortable/able to take part in what was offered?

- ○ Yes
- ○ No
- ○ Don't know

Please explain your answer, if you would like to.

Do you think your child's contact with CAMHS helped to improve their mental health?

Answer

N/A don't know ○

What do you think has been the most difficult/challenging thing in accessing CAMHS mental health support for your autistic child?

What do you think is the most positive/helpful thing you have experienced in CAMHS?

Part 4

The final questions ask for your opinions on how autistic children and young people can be better supported with mental health difficulties moving forwards. Please feel free to provide as much or as little detail as you wish.

What support would you like to see for autistic children and young people to help their mental health **generally**?

What do you think should be put in place to better support autistic children and young people with mental health difficulties **accessing CAMHS**?

Is there anything else you think we should know about you or your child’s experiences?

We are currently writing an application for research funding to understand the ways that mental health services can be improved for autistic children and young people. To make sure that we best meet the needs of autistic children and young people, we are looking for your views on what we should prioritise.

What do you think **research** should focus on in terms of good mental health services for autistic children and young people?
